# Light sensitivity of the circadian system in the social Highveld mole-rat *Cryptomys hottentotus pretoriae*

**DOI:** 10.1242/jeb.247793

**Published:** 2024-09-24

**Authors:** Pauline N. C. Chanel, Nigel C. Bennett, Maria K. Oosthuizen

**Affiliations:** ^1^ Department of Zoology and Entomology, University of Pretoria, Private Bag X20, Pretoria 0028, South Africa; ^2^ Mammal Research Institute, Department of Zoology and Entomology, University of Pretoria, Pretoria 0002, South Africa

**Keywords:** Activity, Circadian rhythm, *Cryptomys*, Light intensity, Mole-rat, Visual system

## Abstract

Highveld mole-rats (*Cryptomys hottentotus pretoriae*) are social rodents that inhabit networks of subterranean tunnels. In their natural environment, they are rarely exposed to light, and consequently their visual systems have regressed over evolutionary time. However, in the laboratory they display nocturnal activity, suggesting that they are sensitive to changes in ambient illumination. We examined the robustness of the Highveld mole-rat circadian system by assessing its locomotor activity under decreasing light intensities. Mole-rats were subjected to seven consecutive light cycles commencing with a control cycle (overhead fluorescent lighting at 150 lx), followed by decreasing LED lighting (500, 300, 100, 10 and 1 lx) on a 12 h light:12 h dark (L:D) photoperiod and finally a constant darkness (DD) cycle. Mole-rats displayed nocturnal activity under the whole range of experimental lighting conditions, with a distinct spike in activity at the end of the dark phase in all cycles. The mole-rats were least active during the control cycle under fluorescent light, locomotor activity increased steadily with decreasing LED light intensities, and the highest activity was exhibited when the light was completely removed. In constant darkness, mole-rats displayed free-running rhythms with periods (τ) ranging from 23.77 to 24.38 h, but was overall very close to 24 h at 24.07 h. Our findings confirm that the Highveld mole-rat has a higher threshold for light compared with aboveground dwelling rodents, which is congruent with previous neurological findings, and has implications for behavioural rhythms.

## Teaser

**Summary:** Day-time light suppresses locomotor activity of Highveld mole-rats in a dose-dependent manner, confirming that this species has a higher threshold for light responsiveness compared with aboveground rodents.

## INTRODUCTION

Biological rhythms are ubiquitous in nature, with animals exhibiting circadian, circannual and circatidal rhythms under natural conditions (Kumar, 2017). Many of these rhythms are endogenously generated by internal biological clocks. Biological rhythms are believed to provide an adaptive advantage to organisms as they allow organisms to anticipate environmental changes rather than react to them (Krittika and Yadav, 2020).

Circadian rhythms are the best studied of biological rhythms. Circadian rhythms are innate, predictable, biological cycles resulting from internal biological clock genes that, when synchronised to external light cycles in particular or, less frequently, other cyclic cues, give rise to diel rhythms in organisms (Aschoff, 1964; Kumar, 2017).

The period or length of circadian rhythms is inherited, and both the period and oscillation of the amplitude are genetically determined (King and Takahashi, 2000; Lowrey and Takahashi, 2011). In the absence of external signals, circadian rhythms will free-run with an innate period of approximately 24 h, hence the term circadian (‘circa’, around and ‘diem’, a day) (Takahashi et al., 2008). Free-running rhythms represent the inherent rhythm under constant conditions (Schöttner et al., 2006).

As circadian rhythms are not exactly 24 h long, they must be synchronised to the external environment. Entrainment is the daily phase-shifting of circadian rhythms in response to environmental cues and is maintained by a harmonious interaction of the endogenous rhythms and environmental conditions (Golombek and Rosenstein, 2010). These environmental zeitgebers include light, temperature and feeding times amongst others (Aschoff, 1960; Golombek and Rosenstein, 2010). The light/dark cycle is the most predictable and preferred environmental cue, and therefore provides the strongest signal for entrainment (Gutjahr et al., 2004; LeGates et al., 2014; Vasicek et al., 2005). Zeitgebers trigger the master circadian clock that controls entrainment to the external environment and synchronises the peripheral oscillators in other regions of the organism (Aschoff, 1960; Schibler et al., 2015).

The circadian clock is located in the suprachiasmatic nucleus (SCN), a paired structure in the basal hypothalamus, comprising of some 10,000 cells each (Hastings et al., 2018; LeGates et al., 2014). Circadian rhythms are generated by endogenous oscillators and manifested as behavioural and physiological processes (Schwartz and Klerman, 2019). These daily rhythms affect processes such as alertness, performance, the endocrine system, blood pressure, body temperature as well as locomotor activity (Browman, 1937; Kleine and Rossmanith, 2016; Schwartz and Klerman, 2019; Wurtman, 1975). Entrainment of the circadian clock to external cues allows organisms to regulate their biological and physiological processes according to both the day and season, providing the organism with the ability not only to adapt to but also to predict changes in the environment (Benstaali et al., 2001; Gwinner, 2012).

In mammals, light can only reach the circadian clock through the eyes via the retinohypothalamic tract (Hastings et al., 2018; Lucas et al., 2012). The retina contains photoreceptive pigments in the cones, rods and retinal ganglion cells (RGCs) that are sensitive to and absorb light (Hecht, 1937; Lucas et al., 2012; Van der Merwe et al., 2018). Cones and rods are primarily involved in vision, whereas RGCs contain photopigments known as melanopsins that are involved in circadian functions (Do, 2019). RGCs can be activated by many wavelengths of light, but they are primarily excited by blue light, with their peak activity at 480 nm (Mure et al., 2007). The light signal travels from photoreceptors in the retina through the retinohypothalamic tract, a subset of axons from the intrinsically photoreceptive RGCs, to the SCN (Hastings et al., 2018; Moore and Lenn, 1972). Disruption of the circadian clock can have direct effects on the survival of animals; for example, chipmunks which had their SCN lesioned exhibited irregular night-time activity that subsequently resulted in increased predation by weasels (DeCoursey et al., 2000).

The Highveld mole-rat (*Cryptomys hottentotus pretoriae*) is a social, completely subterranean rodent (Haupt et al., 2017). Under laboratory conditions, the Highveld mole-rat is nocturnal (Oosthuizen et al., 2003); however, it appears that their activity is more variable under natural conditions in their burrows (Oosthuizen et al., 2021). Highveld mole-rats live in colonies of between 2 and 12 individuals in the Highveld of South Africa (Janse van Rensburg et al., 2002). Its subterranean environment provides relatively constant ambient temperatures and protection from above-ground predators; however, it also presents several challenges (Bennett et al., 1988; Burda et al., 2007). In their burrows, mole-rats are hardly, if ever, exposed to light, oxygen concentrations are low (hypoxic) while CO_2_ concentrations are high (hypercapnic), and the relative humidity is high (Bennett and Faulkes, 2000; Burda et al., 2007). Mole-rats are very well adapted to their subterranean niche and have several morphological and physiological adaptations to it. Their eyes are small (microphthalmic) and retain little visual acuity; however, they are able to detect changes in the form and brightness of light (Němec et al., 2008; Oosthuizen et al., 2005; Oosthuizen and Bennett, 2022). Nevertheless, the subspecies contained in the genus *Cryptomys* have small lenses, which suggests an adaptation for high light intensities (Peichl et al., 2004). In the eye, the rod-dominated retina allows for the reception of low light intensities and, interestingly, they also have a significant population of short wavelength cones (Cooper et al., 1993; Němec et al., 2008). This suggests that the eyes of African mole-rats are more similar to those of diurnal surface dwellers than to other nocturnal rodents (Peichl, 2005). Nevertheless, immunocytochemistry studies assessing neuronal activity in the SCN in response to light indicate that higher light intensities and longer light durations elicit higher responses to light (Oosthuizen et al., 2005, 2010). Given the seemingly contrasting nature of mole-rat vision, we aimed to elucidate the sensitivity of the circadian system of the Highveld mole-rat to light by investigating their locomotor activity.

A study conducted by Oosthuizen et al. (2003) showed that the Highveld mole-rat can entrain its activity in response to changes in the light environment, and thus it has a functional circadian system. We aimed to assess the robustness of the circadian system by investigating the entrainment capacity of the mole-rats in response to decreasing light intensities. We expected the animals to have a clear distinction between day and night activities at higher light intensities, whereas at lower light intensities, we anticipated the activity pattern of *C. h. pretoriae* to become more ill-defined and less robust. We predicted that the animals would display their innate circadian rhythms when subjected to constant darkness (DD), and that they will show a free-running rhythm (τ) of close to 24 h long.

## MATERIALS AND METHODS

### Animal maintenance

Fourteen Highveld mole-rats (*Cryptomys hottentotus pretoriae* Roberts 1913), 10 females (mean±s.e.m. body mass 119.49±19.98 g) and 4 males (126.69±12.97 g), were used in this experiment. The animals originated from laboratory colonies at the Department of Zoology and Entomology, University of Pretoria. The mole-rats had been in the laboratory for about 2 years prior to the experiment and were acclimated to laboratory conditions. Animals were maintained in a climate-controlled animal room on a 12 h light:12 h dark (L:D) cycle at 25°C. The animals were housed individually in plastic containers (58×38×36 cm) lined with wood shavings. All animals were provided with tissue paper for bedding and a plastic shelter or tube. Animals were fed on sweet potatoes and apples every second day at different times of the day to avoid entrainment to feeding times. At feeding times, the general health of the mole-rats was monitored, and cage hygiene was maintained. Mole-rat cages were cleaned, and wood shavings were replaced between the light cycles. Experimental procedures were approved by the Animal Ethics Committee of the University of Pretoria (NAS157/2020).

### Activity recording

Each cage was fitted with an infrared motion detector [Quest PIR internal passive infrared detector; Elite Security Products (ESP), Electronic Lines, London, UK]. The infrared motion detectors were set up over each container to cover the entire floor space to track the activity of the animals during the experiment. Every minute, the recorded activity data were routed to a computer located outside the experimental room and captured by the program VitalView (VitalView^TM^, Minimitter Co., Sunriver, OR, USA).

### Experimental protocol

Animals were exposed to varying light intensities during the day under a 12 h L:12 h D cycle, with light hours between 06:00 h and 18:00 h, for the duration of the experiment. LED strip lights (Inversiones Meseta S.A.C., Lima, Peru) (Fig. S1) connected to a dimmer (LED Dimmer 12–24 V 8A 96-192W, Communica, Pretoria, South Africa) were fitted over the experimental cages to control the light intensity. The animals underwent a control period lasting 1 month and were subsequently subjected to six different light cycles, each lasting 4 weeks. During the control period, the mole-rats were subjected to the standard overhead fluorescent laboratory lights with a light intensity of about 150 lx to obtain a baseline for activity measurements. During the experimental cycles, the light source was changed to LED strip lights (12 V/DC 3528ek h) that were mounted over the animal cages. The first experimental light cycle consisted of a light intensity of 500 lx, followed by 300, 100, 10, 1 lx and constant darkness (DD), respectively. During the DD cycle, feeding, cleaning and health maintenance occurred using a head lamp with a dim red light (light intensity <1 lx) to prevent disruption of the light cycle as Highveld mole-rats lack the appropriate photoreceptors to detect red light. Experimental cycles had a duration of 1 month (4 weeks): the first week was regarded as an entrainment phase, and the last 3 weeks (21 days) were analysed.

### Statistical analysis

Activity data were prepared for analysis in Microsoft Excel (version 16.53, 2021). The 1 min data bins were used to generate double-plotted actograms in the program ActiView (ActiView^TM^, Minimitter Co.) to illustrate activity of the animals for consecutive days of each light illumination exposure. Thereafter, the data counts were summed per hour for analysis in SPSS. The data were not normally distributed; hence, non-parametric statistical tests were carried out using SPSS Statistics 27.0 (SPSS Inc., Chicago, IL, USA). Generalised linear mixed models were used to analyse the data, which controlled for repeated measures and non-parametric data. ID was used as a random factor and different days as repeated measures. We used a gamma distribution with a log link to analyse how total activity and distribution of activity over the 24 h days differed between the different light cycles. Light phase (light/dark) and light cycle were used as fixed factors and we used least significant difference *post hoc* tests. As the sexes were not distributed equally and activity of males and females was overall not significantly different (*P*=0.581), sex was not considered further for interactions. The period of the circadian rhythm of each animal during DD was determined using the Chi-square periodogram function within the Clocklab software (Clock- Lab™; Actimetrics, Evanston, IL, USA). The subjective hour length was calculated for each animal by dividing the individual periods by 24. The subjective hour length per animal was used for analysis and figure illustrations for the DD period. Activity start and end times were not precise enough to determine onsets and offsets; therefore, that was also not considered. A cosinor analysis was performed, using the cosinor program from the Circadian Rhythm Laboratory, to determine the amplitudes and acrophases of the rhythms of each mole-rat during each light cycle. A one-way ANOVA was performed to compare the amplitudes and acrophases of the rhythms between the different light cycles. Statistical significance was maintained at *P*<0.05.

## RESULTS

### Activity levels with decreasing light intensities

Light intensity had a significant effect on the locomotor activity of the mole-rats (*F*_6,39278_=117.27, *P*<0.001). During the control cycle, mole-rats displayed relatively low activity compared with that during the experimental cycles. Activity levels did not differ significantly during the control cycle and 500 lx, and control cycle and 100 lx; activity for all other light intensities was significantly higher compared with the control. The level of mole-rat activity increased significantly as the light intensity decreased, except for a slight decrease in activity at 100 lx, and at 10 lx and 1 lx where mole-rats exhibited a similar level of activity. Their locomotor activity was the highest during constant darkness (Tables 1 and 2).

**Table 1.**
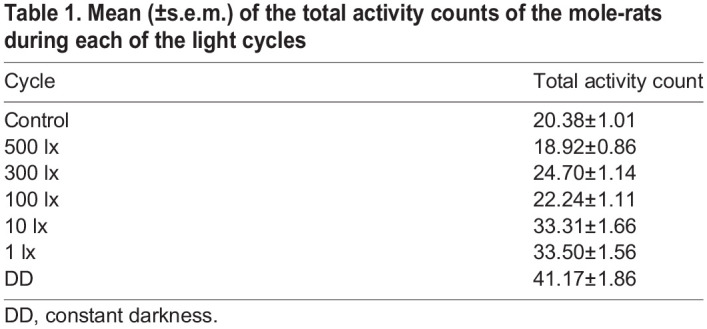
Mean (±s.e.m.) of the total activity counts of the mole-rats during each of the light cycles

**Table 2.**
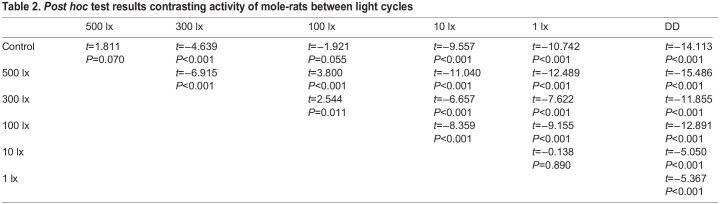
*Post hoc* test results contrasting activity of mole-rats between light cycles

### Entrainment of locomotor activity

**Fig. 1.**
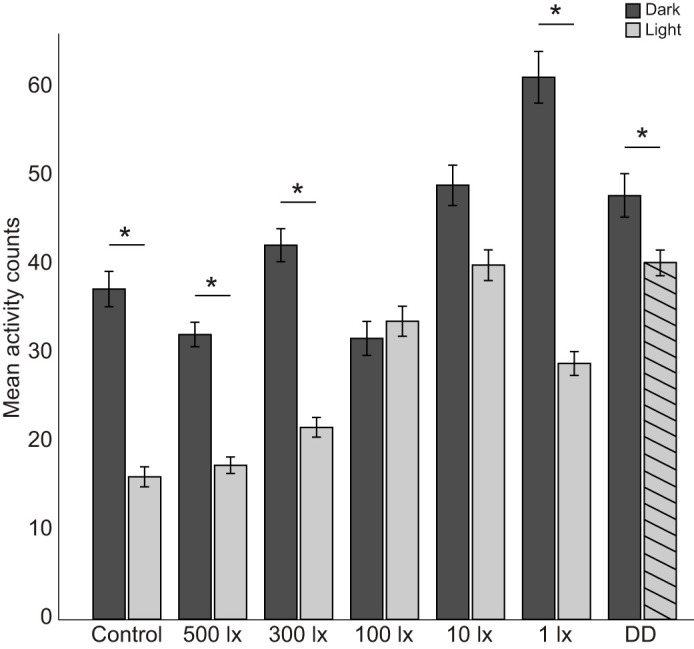
**Mean activity counts (±s.e.m.) for all Highveld mole-rats during the light and dark periods for all light cycles.** For the constant darkness (DD) cycle, the subjective day bar is hatched to illustrate that there was no light present during this period. *N*=12–14, *P<0.05.

Overall, the mole-rats entrained their activity to each light cycle; however, temporal distributions varied between individuals. The animals were more active during the night than during the day (*F*_1,39278_=245.72, *P*<0.001). However, this was not the case for all light cycles (Table 1). Nocturnal activity was higher than diurnal activity for the following cycles: control, 500 lx, 300 lx, 1 lx and subjective night activity for DD (*P*<0.001 for all), and nocturnal and diurnal activity were not significantly different at 100 lx (*P*=0.188) and 10 lx (*P*=0.126; Fig. 1).

### Daily rhythmicity in mole-rats

**Fig. 2.**
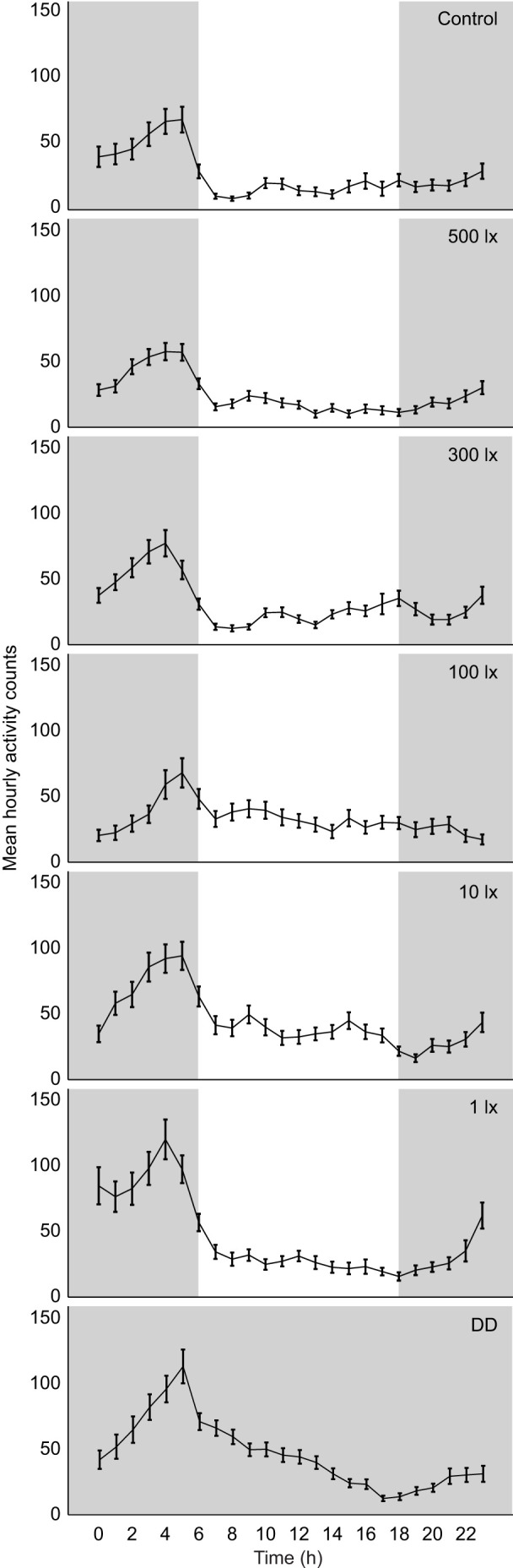
**Mean hourly activity counts (±s.e.m.) over 24 h for all light cycles.** The hourly value was the mean of all animals for that hour averaged over the days of activity that were analysed. The subjective hour length for DD was calculated per animal and the activity values for all animals were averaged in a similar way to the other light cycles. Periods of darkness (night) are indicated in grey. *N*=12–14.

Strong trends were apparent in the overall locomotor activity of the mole-rats with the different light cycles and temporal distribution. Overall, mole-rats appeared to have a peak in activity in the early morning before lights were switched on, accounting for the more nocturnal activity of the animals, whereas lower levels of activity were exhibited during the day activity (Fig. 2; Table S1). The amplitudes of the rhythms did not differ significantly between light cycles (*F*=1.951, *P*=0.161). The acrophases of the rhythms also did not differ between the cycles (*F*=1.794, *P*=0.111).

**Fig. 3.**
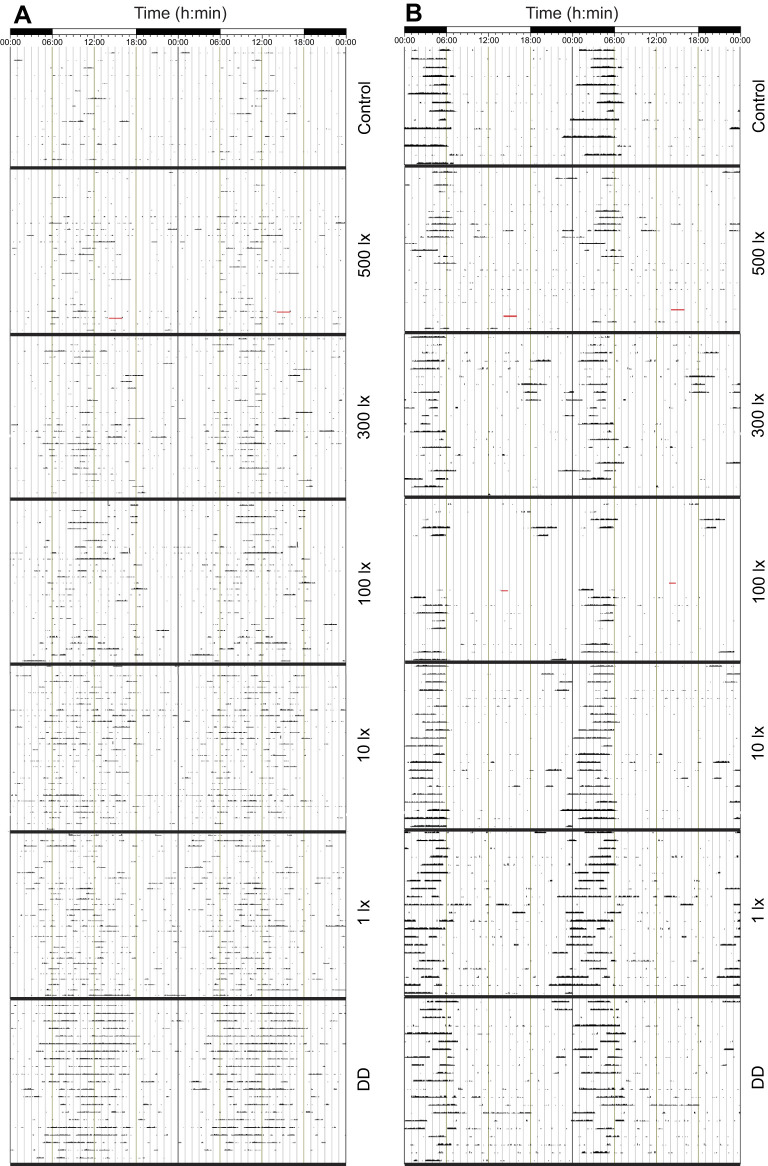
**Double-plotted actograms of locomotor activity over the entire experimental protocol in two Highveld mole-rat females.** (A) An example of a diurnal animal (*C. h. pretoriae* 5) that shows that the mole-rat became more active as the light intensity diminished. (B) An example of a nocturnal animal (*C. h. pretoriae* 6) with distinct activity at the end of the night. Black and white bars at the top of the actogram represent dark and light phases of the light cycle, and each line on the *y*-axis is a consecutive day of activity.

**Fig. 4.**
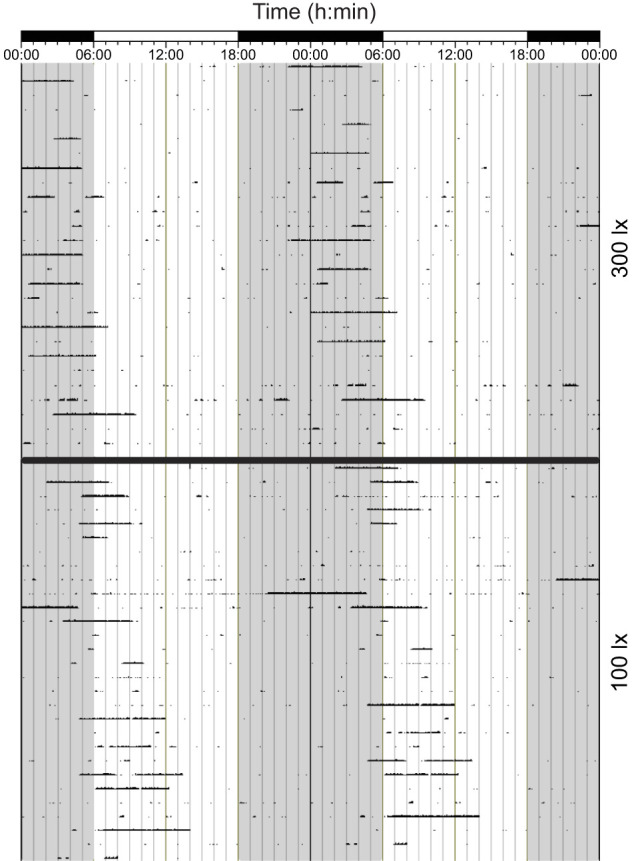
**An actogram of an animal that shifted its activity from nocturnal to diurnal from the 300 lx to 100 lx cycles.** Data are for animal *C. h. pretoriae* 10. Black and white bars at the top of the actogram represent dark and light phases of the light cycle, and each line on the *y*-axis is a consecutive day of activity. For ease of reference, the dark phase is shaded.

However, individual mole-rats showed variable degrees of rhythmicity over the course of the experiment, in terms of both robustness of the rhythm and temporal distribution of the activity (Fig. 3). More animals showed nocturnal activity during the control cycle and higher light intensities compared with lower light intensities. During DD, half of the animals showed more activity during the subjective night phase. Animals remained entrained to the light cycles for the duration of the experiment. Some animals also switched their activity from primarily nocturnal to diurnal (Fig. 4). When subjected to the DD cycle, the period of the endogenous rhythms of the mole-rats was slightly longer than 24 h at 24.07±0.19 h. Six mole-rats had endogenous rhythms longer than 24 h, five mole-rats had endogenous rhythms shorter than 24 h, and one had an endogenous rhythm close to 24 h. There was a general trend in higher levels of activity as the light intensity was lowered (Fig. 3).

## DISCUSSION

Mole-rats of the family Bathyergidae are subterranean rodent moles that live in burrows where they are rarely, if ever, exposed to light. As a result, they have a regressed visual system and microphthalmic eyes (Oosthuizen et al., 2003). Regardless of these features, many species of this family have been investigated under laboratory conditions and were found to possess intact circadian systems, particularly under light:dark cycles and some under ambient temperature cycles (Ackermann et al., 2017; De Vries et al., 2008; Gutjahr et al., 2004; Hart et al., 2004, 2021; Haupt et al., 2017; Oosthuizen et al., 2003; Oosthuizen and Bennett, 2015; Riccio and Goldman, 2000a,b; Schöttner et al., 2006; Van Jaarsveld et al., 2019; Vasicek et al., 2005). We assessed the sensitivity of the circadian system of Highveld mole-rats by examining their locomotor activity under decreasing day-time light intensities.

Highveld mole-rats were able to entrain well to light cycles, maintaining a constant period of 24 h during all experimental cycles when they were exposed to light. Under constant darkness (DD), the circadian periods were close to 24 h, but varied slightly between individuals. This is similar to what has been found for this species previously (Haupt et al., 2017; Oosthuizen et al., 2003), and confirms a functional circadian system in the Highveld mole-rat despite their regressed vision and dark natural habitat.

The light intensities that mole-rats were exposed to altered their locomotor activity. Surprisingly, the activity levels of the mole-rats increased and became more robust with decreasing day-time light intensities, with the highest level of activity exhibited under constant darkness. This is in contrast with the Cape mole-rat, a solitary mole-rat species that appeared unresponsive to light intensity. Adult Cape mole-rats displayed constant levels of locomotor activity despite changes in light intensity in a similar experimental setup (Braunstein et al., 2023). During the control cycle, mole-rats were subjected to standard laboratory conditions with fluorescent overhead lights with an intensity comparable to that of the higher experimental LED light intensities. During the day, there is a greater amount of light available, and the primary photoreceptor used for vision in diurnal animals, the cones, require higher light intensities for activation (Conner, 1982). Similarly, the photoreceptive pigments involved in circadian function also appear to be less responsive to light in diurnal compared with nocturnal animals (Sonoda et al., 2020; Schoonderwoerd et al., 2022). In the diurnal four-striped grass mouse (*Rhabdomys pumilio*), subjected to a similar lighting regime, the activity of the mice increased with increasing light intensities (Van der Merwe et al., 2017). This conforms to the notion that they are less sensitive to light and require a greater amount of light to activate the circadian system. The nocturnal Namaqua rock mouse (*Micaelamys namaquensis*), subjected to a similar light regime, increased activity with increasing light intensities (Van der Merwe et al., 2017). Although the Highveld mole-rat displayed primarily nocturnal activity in our study throughout the experimental treatments, the higher activity at lower light intensities also suggests a reduced sensitivity to light.

The LED strip lights used in this experiment show a distinct peak in the blue spectrum around 440 nm, and this peak is evident at higher light intensities, not at 10 and 1 lx. It is also absent from the fluorescent overhead lights that were used during the control phase (Fig. S1). The photoreceptive pigment melanopsin, which informs the circadian system, has been reported to show a peak absorbance in the range of 424 nm (Newman et al., 2003) to 500 nm (Walker et al., 2008). The presence of this peak in the blue spectrum of light may reinforce the effect of light on the circadian systems of the mole-rats and suggests that both the intensity of the light and its spectral distribution may be important for the behavioural responses of the animals.

Light also regulates melatonin secretion (Gutjahr et al., 2004). Melatonin is produced by the pineal gland at night, and its secretion is inhibited by light (Arendt, 2005). Despite the regressed ocular systems of mole-rats, the pineal–melatonin pathway still responds to photoperiod (Green and Romero, 1997). A study on the Mashona mole-rat revealed that light reduced activity under a masking photoperiod (Vasicek et al., 2005). A similar effect is found in aboveground dwelling, nocturnal mammals (Mrosovsky, 1999; Shuboni et al., 2012; Viljoen and Oosthuizen, 2023). Melatonin secretion is subject to after-effects (Karp et al., 1990) and this is also true for animals living in environments devoid of light. Gutjahr et al. (2004) found that light intensities as low as 13.66 lx were sufficient to lower plasma melatonin concentrations in Highveld mole-rats. Exposure to long periods of light when mole-rats would usually not be exposed to light has an effect lasting into the night, thus reducing their overall activity (Hart et al., 2004). This effect offers a plausible explanation for the increase in activity levels at light intensities of 10 lx and below in the Highveld mole-rat. In nocturnal rodents, melatonin was found to abnormally increase activity and alertness at night (Bilu and Kronfeld-Schor, 2013) and to disrupt sleeping patterns (Gandhi et al., 2015). Several other experiments observing the activity of Wistar rats when exposed to light corroborated this finding (Molcan et al., 2019; Opperhuizen et al., 2017; Zeman et al., 2016).

In their natural habitat, mole-rats are not exposed to light frequently, and they probably do not require a high sensitivity to light for their daily activities, but the ability to perceive light may still be important. Highveld mole-rats dig rather shallow burrows (roughly 20 cm below ground), increasing the possibility that they expose themselves to light while digging and foraging (Bennett et al., 1988). This brief exposure to light may aid the mole-rats in resetting their circadian rhythms (Gutjahr et al., 2004). Vision and light perception in the African mole-rats is also thought to be linked to anti-predator behaviour and repair of open sections of their burrows (Němec et al., 2008). Mole-rats probably use other cues for seasonal synchronisation of their rhythms as they are not exposed to light regularly and consistently enough to provide useful photoperiodic information.

There appear to be species differences in mole-rats regarding the sensitivity to light that are related to the social structure of the animals. Cape mole-rats that were subjected to a similar experimental protocol did not alter their activity in response to a range of light intensities (Braunstein et al., 2023). This suggests that the circadian system of the solitary species is more sensitive to light compared with that of the social species. Previously, we have shown that the SCN of solitary species appears to be more sensitive to light compared with that of social species (Oosthuizen et al., 2005). In the solitary Cape mole-rat, light is integrated effectively in the SCN and neurons are selectively responsive to light according to the time of day, similar to what is observed in surface-dwelling rodents. In contrast, light responsiveness of the SCN neurons in the social Highveld mole-rat was not selective according to the phase of the circadian clock (Oosthuizen et al., 2005). These neurological results appear to correspond to the behavioural results obtained in this study.

### Conclusion

In conclusion, it is evident that Highveld mole-rats possess intact circadian systems that can be entrained to the light:dark cycle. We anticipated that mole-rats would lose entrainment to light at lower intensities; however, they maintained consistent entrainment and became increasingly active. Light appears to mask activity in Highveld mole-rats, and this is likely linked to melatonin. As melatonin and its after-effects seem to change locomotor activity, it appears that light starts to lose its effectiveness on the suppression of melatonin secretion from the pineal gland around 10 lx. This suggests that these social mole-rats have a higher threshold to light compared with other aboveground dwelling rodents, as well as solitary mole-rats. This study provides the first behavioural evidence that subterranean social mole-rats have a higher threshold to light compared with both solitary mole-rats and other aboveground dwelling animals, and that this is reflected in their activity. Nevertheless, it is apparent that both the intensity and the spectral composition of light affect the behavioural responses of the animals, and the spectral composition component warrants further investigation.

## Supplementary Material

10.1242/jexbio.247793_sup1 Supplementary information
