# Supplementary material for: Light sensitivity of the circadian system in the social Highveld mole-rat Cryptomys hottentotus pretoriae
Source: J Exp Biol. 2024 Sep 24;227(18):jeb247793. doi: 10.1242/jeb.247793 (PMC11449439; doi:10.1242/jeb.247793)
Supplement: Supplementary information [file jexbio-227-247793-s1.pdf]

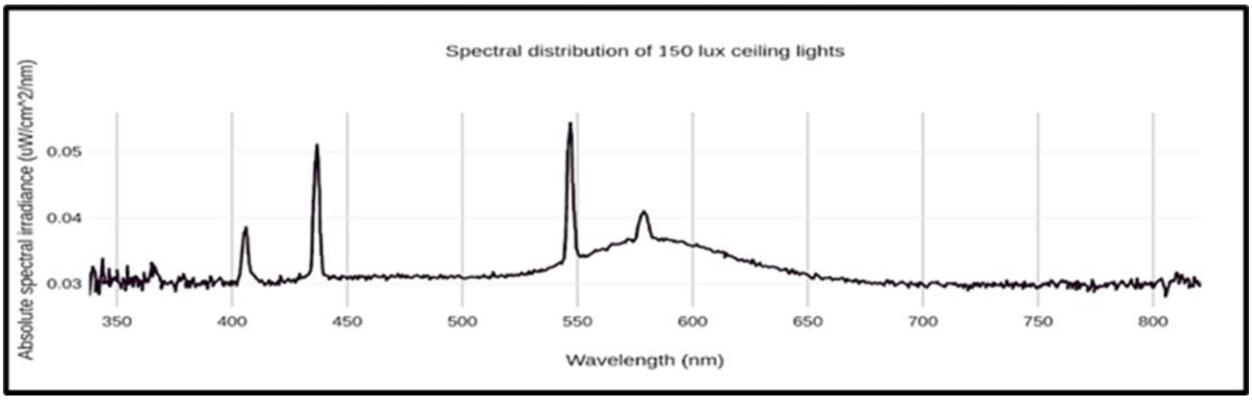
Spectral distribution of 150 lux ceiling lights
Absolute spectral irradiance (uW/cm^2/nm)
0.05
0.04
0.03
350 400 450 500 550 600 650 700 750 800
Wavelength (nm)

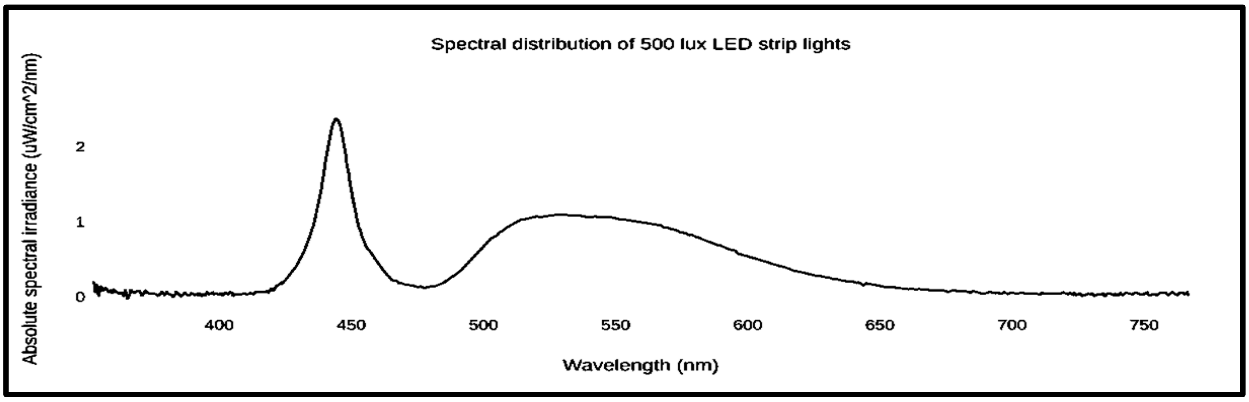
Spectral distribution of 500 lux LED strip lights
Absolute spectral irradiance (uW/cm^2/nm)
2
1
0
400 450 500 550 600 650 700 750
Wavelength (nm)

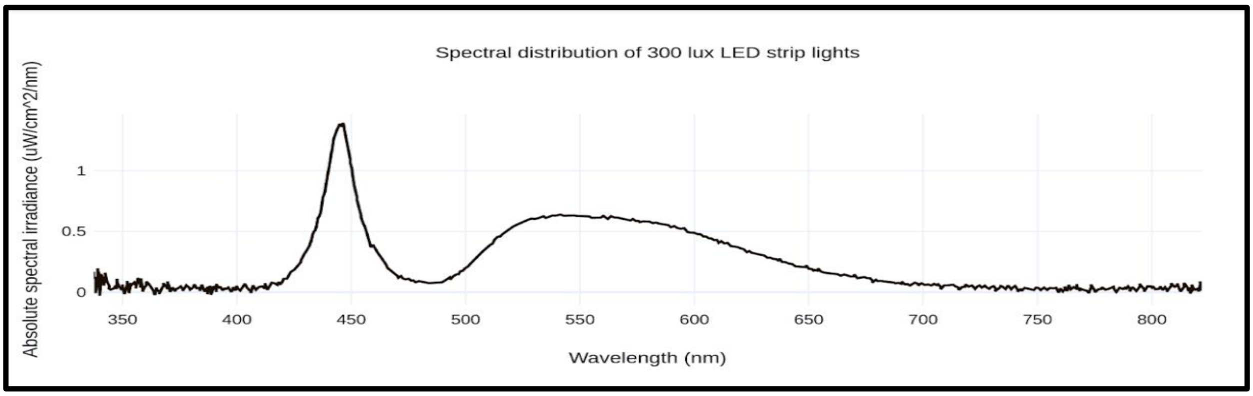
Spectral distribution of 300 lux LED strip lights
Absolute spectral irradiance (uW/cm^2/nm)
1
0.5
0
350 400 450 500 550 600 650 700 750 800
Wavelength (nm)

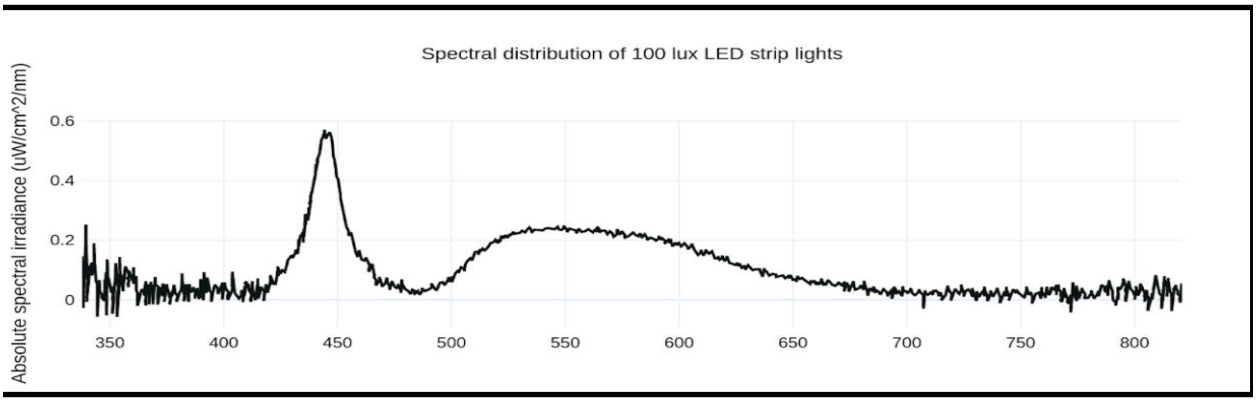
Spectral distribution of 100 lux LED strip lights
Absolute spectral irradiance (uW/cm^2/nm)
0.6
0.4
0.2
0
350 400 450 500 550 600 650 700 750 800

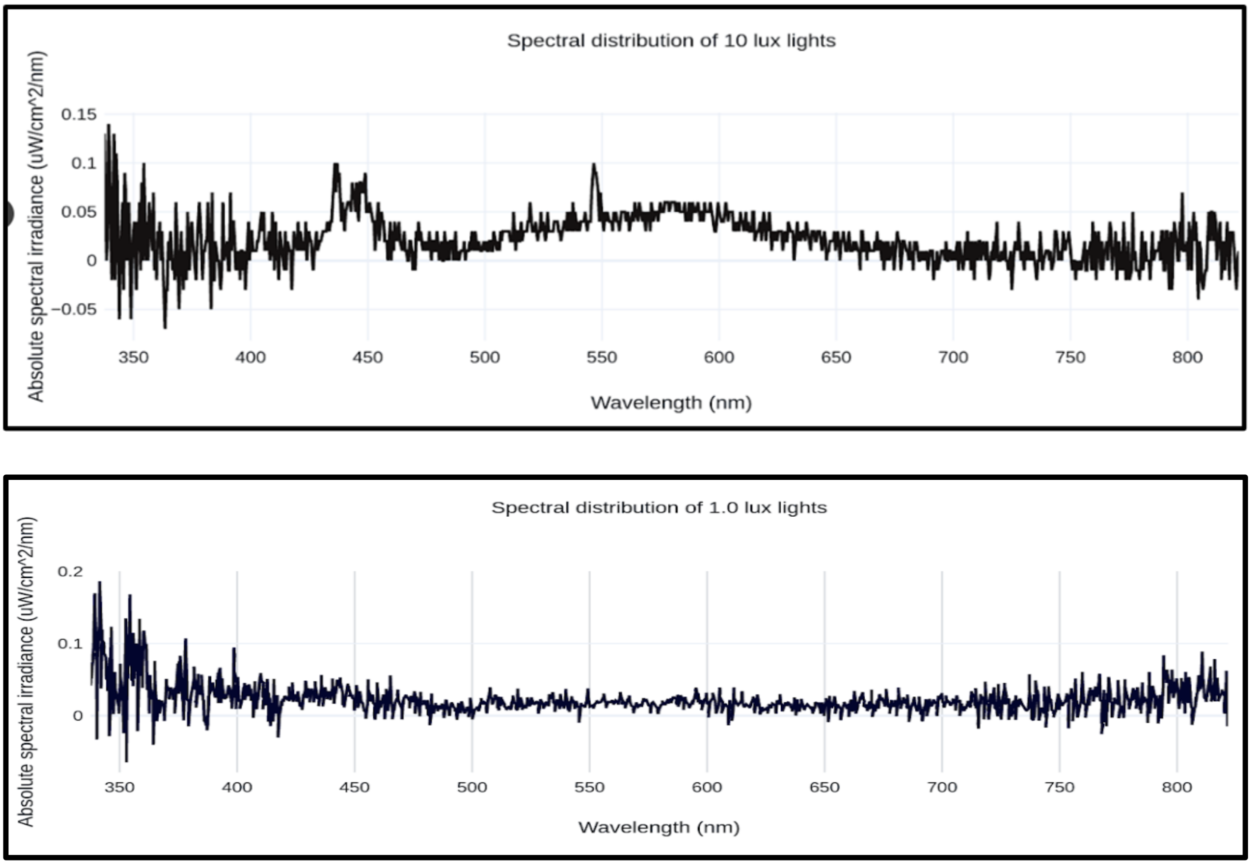

Fig. S1. Spectral distribution of the fluorescent and LED light sources used in the experiment. LED lights were measured at each light intensity

Table S1. Mean (± SE) of the mole-rat activity during the light and dark phases of each light cycle.

Cycle	Phase	Mean (± SE)
Control	Dark	36.93 ± 1.99
	Light	15.91 ± 1.13
500 Lux	Dark	31.84 ± 1.38
	Light	17.20 ± 0.93
300 Lux	Dark	41.84 ± 1.84
	Light	21.44 ± 1.10
100 Lux	Dark	31.40 ± 1.90
	Light	33.33 ± 1.68
10 Lux	Dark	48.56 ± 2.25
	Light	39.61 ± 1.71
1 Lux	Dark	60.66 ± 2.90
	Light	28.60 ± 1.33
DD	Dark	48.12 ± 2.23
	Light	41.89 ± 1.39

Journal of Experimental Biology • Supplementary information
